# Sound and Noise Sources in Sonotubometry: An Investigation of Eustachian Tube Assessment

**DOI:** 10.1007/s10439-024-03526-9

**Published:** 2024-05-13

**Authors:** Tobia Sebastiano Nava, Maximilian Nussbaumer, James R. Tysome, Michael P. F. Sutcliffe

**Affiliations:** 1 Department of Engineering, University of Cambridge, Trumpington St, Cambridge, CB2 1PZ UK; 2 Department of Otorhinolaryngology, Cambridge University Hospitals, Hills Rd, Cambridge, CB2 0QQ UK

**Keywords:** Sonotubometry, Eustachian tube, Digital signal processing, Acoustic modelling

## Abstract

This research aims to enhance the understanding of the acoustic processes occurring during sonotubometry, a method used to assess the Eustachian tube (ET) function. Recent advancements in digital signal processing enable a more comprehensive data analysis. In this project, a silicone model of the ET was developed to systematically study the existing noise and sound sources. These measurements were then compared with recordings from human subjects. Three distinct ’noise sources’ were identified, which can influence the assessment of the ET opening using transmission measurements of the imposed signal: sound leakage from the speaker, a clicking noise at the initiation of ET opening, and rumbling/swallowing noise. Through spectral analysis, it was also possible to ascertain the spectral and temporal occurrence of these sound and noise types. The silicone model exhibited remarkable similarity to the healthy human ET, making it a robust experimental model for investigating the acoustics of sonotubometry. The findings underscore the significance of delving deeper into the analysed sound, as the noise occurring during sonotubometry can be easily misconstrued as an actual ET opening. Particularly, careful consideration is warranted when evaluating data involving clicking and swallowing noise.

## Introduction

The Eustachian tube (henceforth ET) is a small passage connecting the middle ear with the upper part of the throat (nasopharynx) [1]. Its key function is to periodically open up to equilibrate pressure and aerate the middle ear. It is estimated that 0.9 % of the adult population is affected by obstructive ET dysfunction (henceforth OETD), a condition where the ET does not open up adequately [2].

Clinical assessment of OETD remains difficult due to the small size and difficult anatomical location of the ET. To date, there is no gold standard for the assessment of the OETD [3]. Sonotubometry is a technique used to assess the functionality of the ET by applying sound to the nostril and measuring the amount of sound that passes through the ET into the outer ear canal [4]. Compared to other existing methods to assess the ET, sonotubometry does not require an artificial change in middle ear pressure and could therefore be considered more physiologically valid as it does not interfere with the normal ET function.

Many clinical studies aiming to improve sonotubometry have been performed over the years, but the lack of reliability and repeatability mean that it has not gained wider traction as a diagnostic technique [5].

In the past, extensive research was performed to optimise the acoustic data processing and evaluation of sonotubometry [6–8]. However, despite these investigations the results across studies remained inconsistent and thus leave many open questions [4]. Especially, the sound and noise sources present during sonotubometry and how they affect the success of measurements have not been investigated in detail. In addition, the optimal choice of parameters such as sound type and sound amplitude are not well investigated.

The overarching aim of this study is to identify the acoustical processes during sonotubometry. More specifically, one of the objectives is to determine the different noise sources and their impact on sonotubometry. Understanding the impact of the noise sources is critical in order to distinguish ET openings from spurious noise present during sonotubometry.

To achieve this larger aim, the goal is to develop an experimental acoustical model of the ET and use modern digital signal processing. In addition, human measurements are used for comparison and validation. This study could help to improve reliability of sonotubometry and ultimately, its clinical usability.

We developed a custom-built sonotubometer for the purpose of this study and used silicone to construct a model of the nose-ear transmission line. Emphasis was placed on mimicking the acoustic properties of the nose-ear transmission line to study sonotubometry in a controlled environment. Subsequently, we compared the resulting data to human sonotubometry measurements for validation.

## Materials and Methods

In Sect. “Silicone Model” the methods used to build the silicone model are presented and in Sect. “Sonotubometer” the sonotubometer device developed is described. In the last Sect. “Sonotubometry Measurements”, the acquisition procedure for the acoustic data from both the silicone model and humans is described.

### Silicone Model

A silicone model of the ET was created based on 3D imaging and manufactured using 3D printing. A full description of the manufacturing process can be found below. The resulting silicone model was then used for acoustic measurements.

The model of the nose-ear cavity was designed based on a CT scan of a human cadaver specimen and the standardised nose model [9]. The CT scan was made for the purpose of this research and informed the design of the ET and ear cavity. The standardised nose model was used to create the nasal cavity.

The cadaver specimen for the CT scan was obtained from the Human Anatomy Centre of the University of Cambridge for the purpose of this project. The donor of the specimen provided informed consent prior to death for anatomical research under the Human Tissue Act 2004 (University of Cambridge, Licence Number 12146). The CT scan was performed using a Nikon XT 225 ST microCT scanner (resolution 70 $\upmu$m).

The ET of the model was approximated as an oval shaped tube with a height of 6 mm and a width of 1.2mm in an open state. The thickness of the silicone around the ET was chosen to be 3 mm.This created a sufficiently stiff tube while remaining accessible for manipulation (e.g., opening and closing). The approximate acoustical characteristics of the individual segments of the silicone model are listed in Table 1.

**Table 1:** The acoustical characteristics of the silicone model including the characteristic length and the volume of each segment

Anatomical Segment	Characteristic Length ( mm)	Volume (mm^3^)
Ear Canal	24	39000
Eustachian Tube	42	360
Nasal Cavity	136	1500

**Fig. 1:**
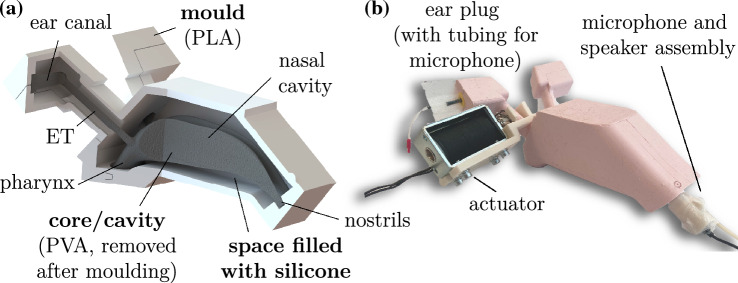
A silicone model of the nose-ear transmission line (approximate dimensions 165 mm$\times$ 60 mm$\times$ 110 mm). Part **a** shows the designed mould, which was 3D printed using PLA. The internal airspace was formed by adding a water-soluble PVA core. Part **b** provides an overview of the setup used to conduct the acoustic measurements, with a speaker inserted into one nostril of the model and a microphone placed in the ear canal. An actuation system was built to create transient ET openings. For more information on the actuation system see Fig. 2

The resulting cavity was 3D printed using an Ultimaker S5 and poly-vinyl alcohol (PVA), which is water-soluble. A mould was designed and also 3D printed using poly-lactic acid (PLA). Two-component silicone with a shore A hardness of 20 (Silastic 3481 RTV Silicone Moulding Rubber, East Coast Fibreglass Supplies) was injected into the mould containing the PVA core using a 60 mL syringe. The silicone was left to cure for several days. The water-soluble core was subsequently removed using hot water, leaving the final silicone model, see Fig. 1. The tympanic membrane was not included into the design of the model as it was not possible to achieve this in a single moulding process.

The resulting silicone model had open ETs at rest. For static measurements the ETs were closed using a clamp. To model the transient behaviour of the opening and closing of the ET an actuation system, based on a relay and a solenoid pin, was built.

**Fig. 2:**
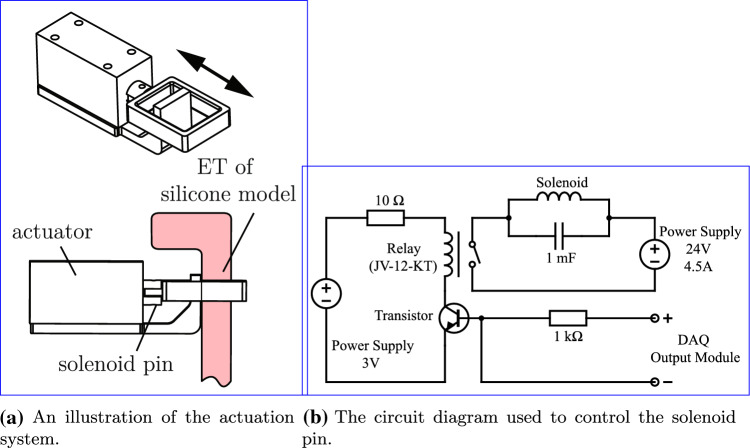
The silicone model was actuated using a solenoid pin. Part **a** shows how the solenoid pin clamped the ET when power was applied to the actuator. The actuation system was used to open and close the ET of the silicone model. Part **b** illustrates the circuit diagram used to control the actuator. A relay was used to control the high voltage circuit of the solenoid through the low voltage circuit from the DAQ. A power supply and transistor amplified the DAQ signal

The solenoid pin was mounted on one of the two identical ET canals using a 3D-printed bracket, see Fig. 2a. The other ET was left open for the entire duration of the experiments. The pin was controlled using a relay (3V Non-Latching Relay, Fujitsu, Tokyo, Japan) connected to a data acquisition (DAQ) device with an analogue output (NI 9260, National Instruments, Texas, USA). The solenoid pin was mounted around the ET of the silicone model, see Fig. 1b. A load was applied to the ET by powering the solenoid pin (RS PRO Linear Solenoid, RS Components LTD., Corby, UK). When the electric current was switched off the pin released and the ET opened. A rubber band was used to dampen and restrict the distance the pin could travel.

### Sonotubometer

**Fig. 3:**
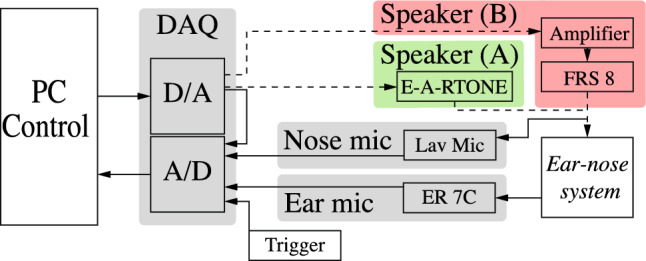
Schematic overview of the sonotubometry device, depicting the flow of the sound created (D/A, digital to analogue) and of the signal recorded (A/D, analogue to digital). The **A** E-A-RTONE speaker was exclusively used for the silicone model and the **B** FRS 8 speaker setup only for measurements in humans

A sonotubometer was built based on the commercially available device, JK-05A RION (Rion Co., Tokyo, Japan). The device developed consisted of a speaker and two microphones. For the measurements with the silicone model, an E-A-RTONE Insert Earphone 3A (3 M, Minnesota, USA) was used, while for the measurements with healthy participants, an FRS 8 (Visatron, Haan, Germany) was used. One microphone (Lavalier Wired Microphone, RS PRO, London, UK) was placed inside the enclosure of the speaker. The whole speaker setup was placed at the nostril. The second microphone (ER-7C Probe Microphone, Etymotic Research, Texas, USA) was placed in the ear canal using suitable foam ear tips to insulate the microphone from ambient noise. The free-field response of two microphones were measured and calibrated according to a predefined 1 kHz test sound. The data was recorded using a DAQ Sound and Vibration Input Module (9234), National Instruments, Texas, USA. A schematic overview of the device can be seen in Fig. 3.

### Sonotubometry Measurements

In the following section the process for acquiring the sonotubometry measurements is elaborated. The method used is similar for humans and for the silicone model. Any differences will be explained in detail.

For all measurements the speaker assembly was placed on the same side as the microphone (i.e. both on the left or right side). Attention was given to creating a good seal in the ear canal to minimise the impact of external noise.

The measurements with the sonotubometer were performed using a customised user interface in Matlab 2021a. For all measurements, either no sound or a white noise signal (125 Hz to 12 800 Hz) was used. The measurements were sampled at a frequency of 25.6 kHz. The data was subsequently analysed using fast Fourier transform and short-time Fourier transforms (STFT) in Matlab. To calculate the STFT a Hanning window was used with a block-size of $2^9$.

The decibel of sound pressure level (dB SPL) was calculated based on the reference sound pressure of $p_0 = {0.02\,\mathrm{\text {m}\text {Pa}}}$. From here onwards ‘dB’ and ‘dB SPL’ will be used interchangably.

For the measurements in humans, specific manoeuvres were used. For prolonged ET opening, mandibular movements (moving the jaw) were used to open the ET [5]. Transient ET openings were achieved using dry swallowing (i.e., without water).

All measurements were performed in a quiet room with minimal acoustic disturbances.

For the purpose of this study a clear distinction was made between sound and noise when analysing the acoustic data. The term ‘sound’ is only used when referring to the sound that was applied to the nostril and subsequently travels through the ET. ‘Noise’, on the other hand, refers to any other form of acoustic signal that occurs during the measurements, e.g., swallowing noise, ambient noise or sound leakage.

## Results

In this section the results from the silicone model and the healthy participants will be presented. Table 2 gives an overview of the sections and how they overlap.

**Table 2:** An overview of the different results sections for the silicone model and the healthy participants and their overlap

	Silicone Model	Healthy Volunteers
Static open and closed	Sect. “Noise Sources in the Silicone Model”	–
Transient ET opening	Sects. “Impact of Sound on the Silicone model”, “Transient Sound and Noise Patterns in Detail”	Sect. “Noise Pattern in Humans”
Scaling of sound level	–	Sect. Scaling of Sound and Noise in Humans

### Noise Sources in the Silicone Model

**Fig. 4:**
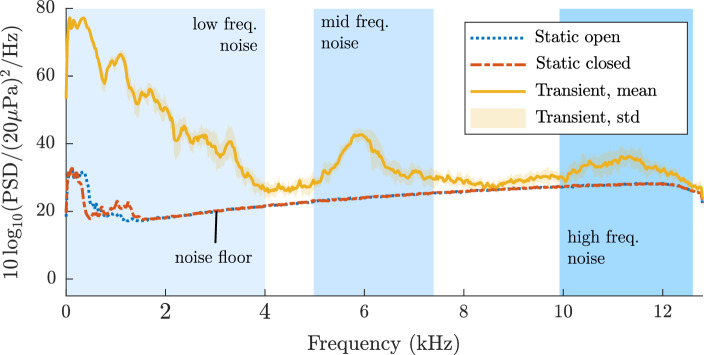
The frequency spectrum of the silicone model of the ET when no sound was applied. Minimal acoustic noise was measured for the statically closed and open case. The measured lines largely follows the free-field response of the acoustic equipment. However, a significant amount of noise can be seen for the transient opening, primarily in the low frequency region. The curves for the static measurements are shown without a standard deviation as they were averaged over a duration of 60s

In the first instance, the noise sources in the silicone model were studied for both the static and transient cases. Figure 4 shows the resulting frequency spectra for a statically closed ET, a statically open ET and a transient opening ET (with an opening duration of 0.5s).

At this stage, no sound was applied to the system, which reveals the noise created by the system itself. For the static cases, the frequency spectrum was calculated over a period of 60 s to minimise the effect of ambient noise sources.

For the transient measurements, seven recordings were performed. The mean and standard deviation of the frequency spectra of those recordings were subsequently calculated. The measurements were truncated based on the synchronised electrical signal provided to the solenoid pin.

Figure 4 illustrates that the curves for the statically open and closed states collapse to the same level. This corresponds to the ambient background noise and the noise floor originating from the electrical components of the sound equipment (e.g. microphone, cables, DAQ).

A considerable amount of acoustic noise (e.g., an approximate increase of 20 dBSPL between 5 kHz and 7 kHz) was measured for the transient opening of the ET in the silicone model. A distinct frequency pattern with a strong increase of sound amplitude between 0 kHz and 4 kHz, as well as between 5 kHz and 7 kHz was measured. A less pronounced increase in sound levels can also be observed between 10 kHz and 12 kHz. The transient noise pattern exhibits little deviation between individual measurements, which is reflected by the low standard deviation.

### Impact of Sound on the Silicone model

**Fig. 5:**
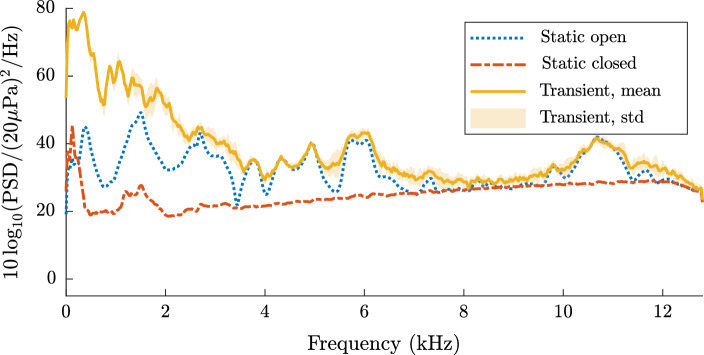
The sound spectrum at the “ear" of the silicone model when applying white noise to the “nose". When the ET is closed, the sound spectrum shows minimal sound penetration. After opening the ET statically, a distinct curve can be observed with clear resonant frequencies and regions of strong attenuation. The transient ET opening further adds noise to the system which results in an additional increase in the sound levels in certain frequency regions despite equal excitation levels

The same measurements as in Sect. “Noise Sources in the Silicone Model” were repeated, with the addition of white noise (approximately 110 dB at the nostril). The resulting frequency spectra can be seen in Fig. 5.

Little change can be seen for the statically closed case between Figs. 4 and 5. A minimal increase in sound was measured between 1 kHz and 2 kHz.

The observed peaks are potentially related to acoustic resonances, however it cannot be excluded that the response shown is due to the specific microphone or speaker characteristics. However, between 7 kHz and 10 kHz, the statically open case overlaps with the statically closed case again, which means that almost no sound penetrated the ET in this frequency range.

For the transient ET opening, little change can be observed between 0 kHz and 4 kHz. This is the region where most noise is generated by the opening mechanism. However, certain peaks can still be observed that are also visible for the statically open case. Overall the sound amplitude for the transient opening is equal to or higher than the statically open case.

### Transient Sound and Noise Patterns in Detail

**Fig. 6:**
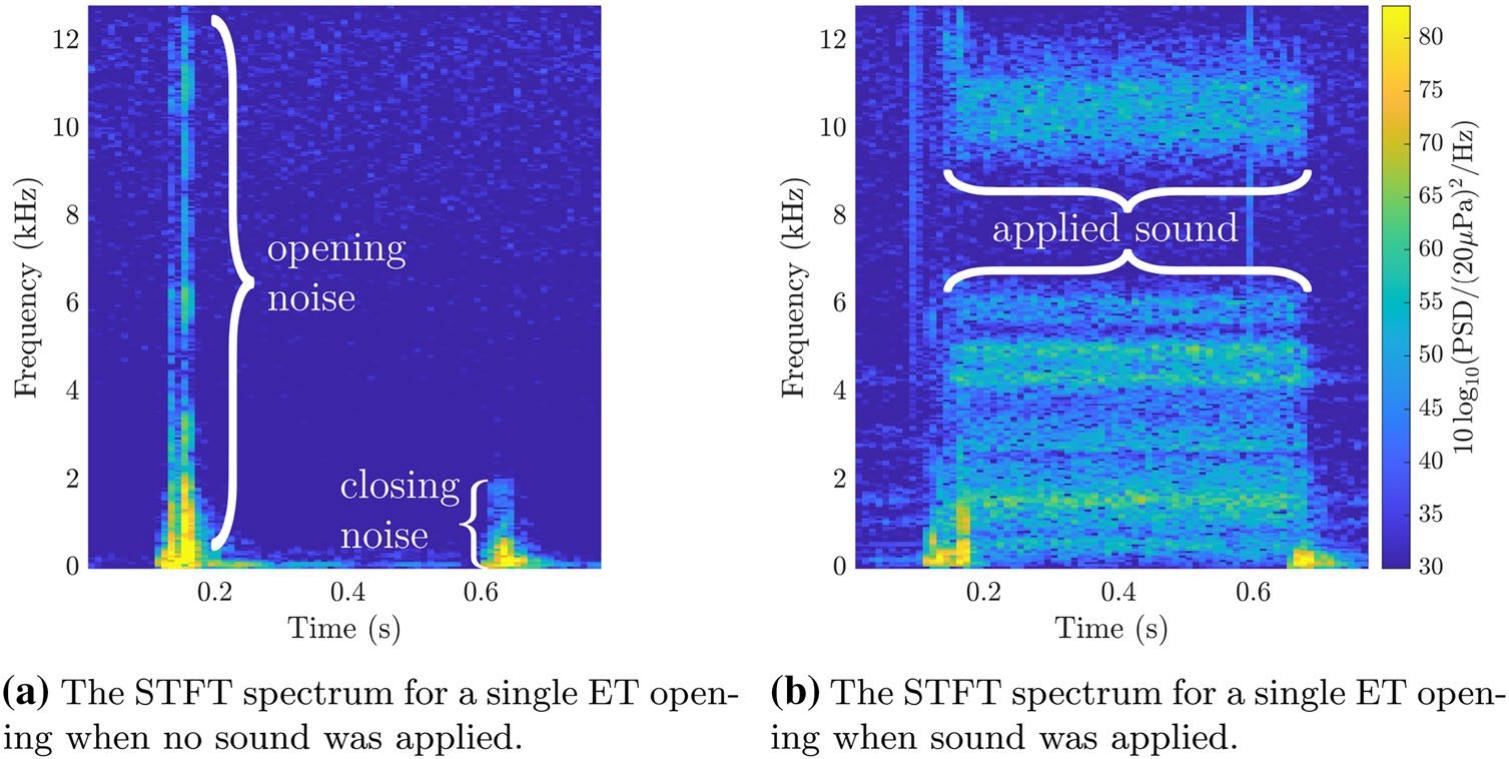
STFT plots of the acoustic signal at the ear of the silicone model for a transient opening ET tube, with **a** no sound applied, **b** 110 dB SPL of white noise sound applied at the nostril. The spectrograms were calculated with a frequency resolution of 50 Hz, 50 % overlap, and a reference pressure of 20e-6 Pa. To simplify the comparison the measurements were synchronised and truncated to 0.8 s

We employed short-time Fourier transforms to investigate the transient opening and closing in more depth. More specifically, the STFTs allow us to identify the contribution of individual noise sources, their frequency components and their distribution over time. Figure 6 shows the created noise from (a) the opening and closing itself, as well as the resulting change when (b) applying sound to the system.

When no sound is applied, a sharp increase in noise of short duration can be observed at 0.2 s. This is the moment when the ET of the silicone model was triggered to transition from the closed to the open state. The recorded noise is present throughout the entire frequency range from 0 kHz to 12.8 kHz and has the perceived characteristics of a clicking noise. Additionally to the clicking noise, a less pronounced increase in sound was also measured at 0.6 s. This was the moment when the ET transitioned back to a closed state.

To assess if the detected noise peaks, as observed in Fig. 6a, do not originate from the actuator itself an additional test was performed without the actuator. In this test the ET was manipulated manually (i.e. pinched and released by hand). While a similar noise pattern was observed upon opening of the ET, the closing noise was not observed during the manual testing of the ET.

When applying sound at the nostril, a similar pattern can be seen for the moment when the ET opened and closed. However, in between those two points, a clear and consistent sound pattern can be seen, similar to the frequency spectrum of the statically open case as seen in Fig. 5. Therefore, there is a clear sequence of when the transient noise (related to the mechanics of the ET opening and closing) and the applied sound penetrating the open ET are present at the ear. This is a feature that was previously not visible by solely looking at the acoustic spectrum of these measurements.

### Noise Pattern in Humans

While it is possible to measure the statically closed ET in humans, it is difficult to measure a statically open ET as it requires a constant actuation of specific muscle groups, which is hard to achieve. Instead, two manoeuvres were used to study the noise patterns in humans, swallowing and mandibular movements. Swallowing is a involuntary reflex which amongst other things also tensions the soft palate, which causes the ET to open temporarily. Mandibular movements target the same muscles but the contraction happens voluntarily without any additional motion or contraction of other muscles.

To compare the noise sources present, similar measurements to those performed on the silicone model were performed on one healthy participant without applying any sound at the nostril. The measurements were performed twice with each manoeuvre being repeated three times for each measurement.

**Fig. 7:**
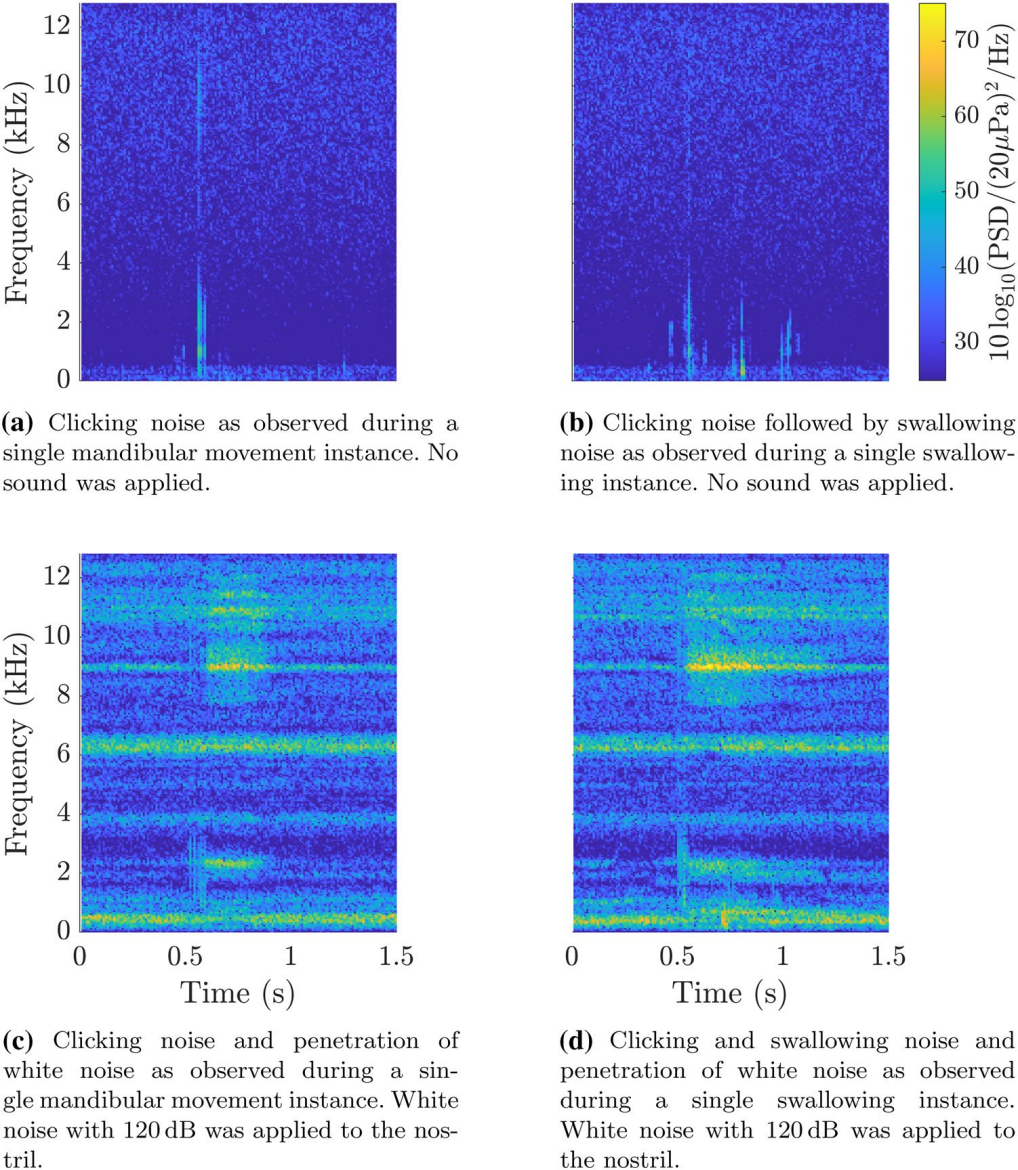
In this series of spectrograms the noise pattern during **a** mandibular movement and **b** swallowing was observed. These measurements were performed in a quiet environment. After applying 120 dB of white noise to the nostril, the effect of the sound during **c** mandibular movement and **d** swallowing was measured. The ET opening occurred at approximately 0.5 s for all cases and the ET closed again at roughly 1.0s. The spectrograms were calculated using a frequency window of 50 Hz, 50 % overlap, and a reference pressure of 20e-6 Pa

Figure 7a shows the characteristic noise pattern when performing mandibular movements. A clear and sharp noise can be measured, which is present in the frequency regions between 0 kHz to 3 kHz and 8 kHz to 11 kHz.

For swallowing (Fig. 7b), a similar sharp increase can be observed. However, this is followed by additional noise in the low-frequency region between 0 kHz to 3 kHz spread over the duration of approximately 0.5 s.

These results indicate that the initial clicking sound, as also seen with the silicone model, is linked to the ET opening itself. However, the additional low-frequency noise seen with swallowing is not directly linked with the ET opening.

### Scaling of Sound and Noise in Humans

To identify the limit at which the applied sound is able to successfully penetrate the ET of a human, one continuous measurement was performed where the applied sound loudness was increased incrementally. This measurement was performed on a single participant. The statically closed state was recorded as well as the transient ET opening using swallowing. Three swallowings were measured at each sound level. Swallowing was chosen as it leads to more consistent results than mandibular movements.

White noise with a sound pressure level ranging from 62 dB to 130 dB was applied to the nostril. 62 dB corresponds to the noise floor (when no sound is applied) expected to originate from the sensor. Each swallowing manoeuvre resulted in an increase in the recorded sound amplitude regardless of the applied sound level from the speaker. This means that the recorded acoustic level increased in amplitude both in the presence and absence of a sound source at the nostril.

A peak in the time domain can commonly be characterised by two basic measurements, the peak height and the peak width. The peak height in this case describes the increase in sound level from the baseline noise level. The peak width on the other hand is the duration of the sound increase, which is commonly associated with the duration of the ET opening.

**Fig. 8:**
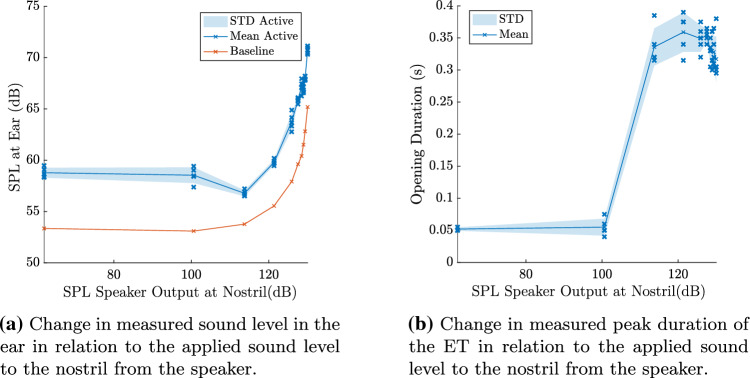
In part **a** the relationship between the applied sound level to the nostril and the recorded sound level at the ear is shown. In part **b** the relationship between the applied sound level and the measured peak width is depicted. ‘Baseline’ refers to the sound level measured in the ear in absence of any manoeuvres. This is averaged over the duration of 10 s. ‘Active’ refers to the sound level measured when performing a manoeuvre

Fig. 8a shows the relationship between the sound level applied by the speaker and the resulting peak height (i.e., the increase of the measured sound level in the ear canal). The resulting peak height was found to be roughly constant between approximately 62 dB and 110 dB.

After this, a sharp increase in recorded sound amplitude was measured between 110 dB and 130 dB roughly following a linear curve. Overall, a higher sound level is needed for measurements in humans in comparison to the silicone model as there is more attenuation present. This is expected to be caused due to the presence of mucus in the ET, higher geometrical complexity and softer walls which dampen the sound signal.

When measuring the peak width (i.e, the duration of the sound increase) in relation to the applied sound level, a sharp rise can be observed with a threshold somewhere between 100 dB and 110 dB, see Fig. 8b. Below 110 dB, the measured peak width was around 0.05 s, which is close to the duration of the clicking as seen before in the spectrograms of the silicone model and the healthy volunteers when the impulsive opening occurred. Above 110 dB the duration was measured to be between 0.3 s to 0.4 s. This is closer to the full opening duration of the ET, which typically lies in the range of 0.3 s to 0.4 s seconds [10, 11].

## Discussion

**Fig. 9:**
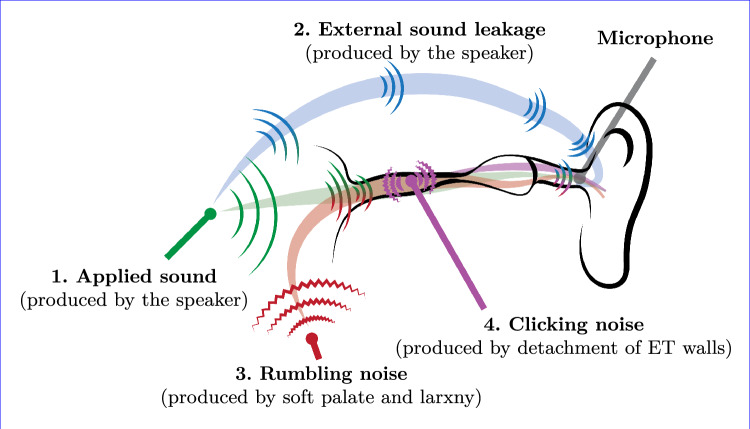
An overview of the identified sound and noise sources measured in the ear canal: the applied sound to the nostril (1), the external sound leakage (2), the rumbling/swallowing noise (3) and the clicking noise from the ET (4)

In the following section, the general acoustic behaviour of the sonotubometry will be discussed. This is followed by an analysis of the identified noise sources and their pathway, starting with the broad frequency clicking noise when opening the ET. Thereafter, the low frequency rumbling noise seen during swallowing will be discussed. An overview is given in Fig. 9. Finally, the importance of the applied sound amplitude is examined.

### Noise sources

When the ET is opened and sound is applied at the nostril there is a clear increase in the sound amplitude at the ear in both the silicone model and in human measurements, as seen in Fig. 5. This highlights the ability of the silicone model to mimic the physiological behaviour of the ET.

For the silcione model we observed that, in contrast to the statically open case, when the ET tube is opened transiently there is a significant increase in the noise level. Similar clicking noise has been reported in the past [12, 13]. However, there has been limited work on assessing its impact on sonotubometry. The measured clicking noise had a distinct pattern which excited almost the entire frequency spectrum. We expect that this noise originates from the ET itself. As the inner walls of the ET in humans are covered by mucus, it will experience surface tension due to moisture. This surface tension is subsequently expected to create the clicking noise upon separation. Therefore, based on the presented results, we assume that the detachment of the inner walls during the opening creates this distinct clicking noise.

When comparing the results of the silicone model with measurements from healthy participants, a similar clicking noise was observed when performing mandibular movements. When performing swallowing, additional swallowing noise was recorded, which primarily occurred in the frequency region from 0 kHz to 4 kHz. While the presence of the swallowing noise has been reported in the past, it was never investigated in its depth [14]. By analysing the spectrogram of the noise and comparing swallows to mandibular movements we were able to characterise the occurrence and frequency components of the noise in more detail. These observations can be applied to dealing with swallowing noise when processing sonotubometry data.

Due to the pronounced and persistent presence of the swallowing noise, we do not recommend this frequency region for the use in sonotubometry, which has also previously been reported using a similar argument [14]. However, they were not able to characterise and identify the origins of these noise sources more closely due to the nonexistence of digital signal processing technology at the time.

In addition to the clicking noise, a small amount of noise was measured in the frequency region of 0 kHz to 2 kHz when closing the ET in the silicone model. However, no equivalent noise was observed when performing measurements on human participants or when stimulating the ET of the silicone model manually. As a result, it is expected that this low frequency noise is an artefact of the actuator design and could stem from the abrupt closing of the ET with the solenoid pin and the resulting vibrations of the ET. As the ear microphone was acoustically shielded using the foam insert, airborne noise is expected to have a lesser impact and the transmission of the closing noise primarily happened through vibration of the silicone model. This is also further supported by the low frequency range of the closing noise.

While performing a mandibular movement creates less noise than swallowing, it is not a feasible method for clinical use, as it requires practice and is less consistent at opening the Eustachian tube (as it is a voluntary movement) [4]. Mandibular movement heavily depends on the muscle tension exerted by the participant which is difficult to control voluntarily. Swallowing, on the other hand, is a reflex that happens involuntarily once triggered, and thus creates more consistent results and is easy to perform [15].

Sound leakage was measured for both the silicone model and in human subjects. This occurs when some of the applied sound leaks to the microphone placed in the ear canal. This is most likely to happen through external sound leakage around the head. While bone or soft tissue conduction is also possible, they are less likely, and their impact would also be smaller [16]. This type of noise does not necessarily affect the measurements as it stays constant throughout the recordings, and the noise floor can be adjusted accordingly.

For both the silicone model and the human subjects, a temporal and spectral separation of the individual noise and signal sources was observed. It was identified that during swallowing the clicking noise occurs first, indicating the start of the opening of the ET. The clicking noise is accompanied by the rumbling noise of the swallowing motion, which is often present until the ET closes again. The applied sound that manages to travel through the ET was always measured after the clicking noise, which further supports the assumption that the clicking is linked to the ET opening. This separation creates the opportunity to optimise the processing and evaluation of the sonotubometry data (e.g., by using bandpass filters) linking the sound more precisely to its source.

### Impact of applied sound level

When studying the relationship between the applied sound and the measured sound level, the results clearly show two regions: below an applied sound level of 110 dB at the nostril, a constant sound level was measured in the ear canal with minimal sound leakage. Above 110 dB, a linear relationship between the applied sound level and recorded sound level was observed.

When looking at the width of the measured sound-increase peaks, a sharp increase was measured at an excitation level of 110 dB where the duration of the peak increases drastically from 0.05 s to 0.35 s. The 0.05 s peak corresponds to the duration of the clicking noise whereas the 0.35 s peak is close to the reported duration of the average ET opening [10]. Therefore, the peak duration appears to be a reliable metric to identify if sound penetrated the open ET or if the measured signal originated from noise.

## Conclusion

Advances in digital signal processing in recent years allow for a more detailed analysis of acoustic data. This created the opportunity to decompose the individual signal and noise sources in more depth than previously possible.

By developing a silicone model, it was possible to create a controlled system to study the acoustics of sonotubometry. The recordings from the silicone model were subsequently validated using sonotubometry measurements from healthy participants. The resulting data suggests that the developed silicone model is capable of mimicking the acoustic properties of the human ET adequately. Furthermore, the data allowed us to identify important noise sources present during sonotubometry.

Three distinct noise sources were identified, clicking noise, swallowing noise and sound leakage.

The presented data supports the theory that the clicking noise originates from the detachment of the surfaces within the ET when opening up, which was reported in the past but was never investigated in connection with sonotubometry [12]. Both the silicone model and the human measurements reliably exhibited this clicking noise upon opening the ET. The silicone model was useful specifically to characterise the clicking noise as it allowed precise control of the opening and closing of the ET and a synchronisation of its actuation with the measured acoustic data which is not possible in humans.

The swallowing noise has a low-frequency spectrum and could be qualitatively described as a “rumbling noise". It is presumed to arise from the movement of the tongue, saliva and larynx. While it is not directly linked to the ET opening, it occurs together as swallowing is the most commonly used manoeuvre for ET opening. Other methods, such as mandibular movement, omit the swallowing noise but are much harder to perform repeatably and are thus not suitable for clinical use.

The relationship between the sound level applied at the nostril and the sound level measured in the ear allowed us to identify the threshold below which the attenuation of the system is too large to reliably measure ET openings. A key observation was that the peak width (i.e., duration of the sound increase) could be a reliable indicator to distinguish noise from true ET openings. This will be addressed in a future study on a larger pool of healthy participants and patients with OETD. Furthermore, the temporal and spectral separation of the signal and noise sources creates an opportunity to further improve sonotubometry and its reliability to detect true ET openings.

Further work will be required to translate these findings into an optimised technique for sonotubometry. This will also require a full assessment of the sensitivity and specificity to assess the usability of sonotubometry as a clinical assessment method for OETD.
